# Health personnel retention strategies in a peri-urban community: an exploratory study on Epworth, Zimbabwe

**DOI:** 10.1186/s12960-016-0113-z

**Published:** 2016-04-27

**Authors:** Bernard Hope Taderera, Stephen Hendricks, Yogan Pillay

**Affiliations:** School of Health Systems and Public Health, University of Pretoria, HW Snyman Building (North), 31 Bophelo Road, Gezina, Pretoria, South Africa; Department of Political and Administrative Studies, University of Zimbabwe, P.O Box MP 167, Mount Pleasant, Harare, Zimbabwe; National Department of Health of the Republic of South Africa, Civitas Building, 222 Cnr Struben St. & Andries St., Pretoria, 0001 South Africa

**Keywords:** Retention, Strategies, Health personnel, Reform, Peri-urban community, Epworth, Zimbabwe

## Abstract

**Background:**

The need to retain health personnel is a policy challenge undermining health system reform of the 21st century. The need to resolve this global health workforce crisis resulted in the First Global Forum on Human Resources for Health in 2008 from which the Kampala Declaration and Agenda for Global Action was formulated. However, whilst there have been several studies exploring the retention of health personnel towards this end, available literature does not provide a detailed narrative on strategies used in peri-urban communities.

The aim of this study was to explore retention strategies implemented in a Zimbabwean peri-urban community between 2009 and 2014 and implications for peri-urban communities towards the health system reform agenda.

**Methods:**

The study was carried out in Epworth, a peri-urban community in Harare, Zimbabwe. The research design was a cross-sectional survey, in which qualitative methods were used in sampling, data collection, reporting and analysis. Qualitative tools were used to collect data through in-depth interviews with purposively selected health personnel managers at 10 local clinics and sample interviews with purposively selected healthcare workers who included registered general nurses, state-certified nurses, midwives, environmental health technicians, nurse aids and community health volunteers at each clinic. Two focus group discussions were carried out with community health volunteers. Qualitative data was subjected to thematic analysis, with coding being performed manually.

**Results:**

A programme-specific strategic partnership between the government and donor community contributed towards the mobilisation of more health personnel, health facilities, worker development and remuneration. To complement this, the Ministry of Health intervened through the review and payment of salaries, support towards post-basic training and development, and protection. The local board, mission and donors contributed through the payment of top-up allowances and provision of non-monetary incentives.

**Conclusions:**

The review of salaries, engagement of international strategic partners, payment of top-up allowances, support towards post-basic training and development, mobilisation of more health personnel, non-monetary incentives and healthcare worker protection were critical towards the retention of health personnel in the Epworth peri-urban community between 2009 and 2014.

## Background

The need to retain health personnel is a policy challenge undermining human resource for health reform and health system appraisal of the 21st century throughout the world [1, 2]. The need to resolve this global health workforce crisis resulted in the First Global Forum on Human Resources for Health in 2008. From this, the Kampala Declaration and Agenda for Global Action was formulated. This declaration and agenda seeks to contribute towards human resource for health reform of post-2008 through, amongst other policy interventions, effective retention of health personnel [3]. The retention of health personnel has favourable implications towards the 2030 Sustainable Development Agenda, particularly goal number 11, aimed at making cities and human settlements inclusive, safe, resilient and sustainable, and goal number 3, towards ensuring healthy lives and promoting well-being for all at all ages [4].

In this regard, whilst there have been several studies exploring retention of healthcare workers around the world, available literature does not provide a detailed narrative of the strategies used in peri-urban communities and the effect that they have on local human resource for health systems of post-2008 [5–8]. Peri-urban communities are an urban fringe between city and countryside characterised by chaotic urbanisation leading to a sprawl. They are a new kind of multifunctional territory and are characterised by the lack of organisational and institutional integration, particularly in developing parts of the world [9]. In Zimbabwe, peri-urban communities have been identified as settlements established in areas that have very small populations but have a potential to grow into bigger urban centres. They have been regularised into devolved local boards, a form of municipal authority that occupy the lowest position in the local governance hierarchy of Zimbabwe. These local boards are established where a centre has peculiar circumstances and where government assistance would be required towards their sustenance. There are four such local boards established at Hwange, Ruwa, Epworth and Chirundu [10]. It is acknowledged that there are different arrangements of peri-urban communities throughout the world.

There were two municipal clinics, one mission clinic and seven private clinics in Epworth. Health personnel at the municipal and mission clinics were managed by the Ministry of Health and Child Care through the Mashonaland East Provincial Medical Directorate (MEPMD) and the Seke District Medical Office (SDMO). It was, however, established that the Epworth Local Board retain ownership of the two municipal clinics and play a limited health personnel management role due to technical and financial incapacity. Health personnel at the seven private clinics were under the management of their owners.

Before 2008, there was only one municipal clinic and one mission clinic serving the community. Epworth experienced a mass exodus of health personnel between 2003 and 2008 owing to the unfavourable macro socio-economic environment that was characterised by a very high rate of inflation. As a result, there were only four nurses and three nurse aides at the mission clinic as outlined in Table 1.

**Table 1:** Health cadres in Epworth before 2007

Facility type	Nursing staff	Other cadres
Municipal clinic	10 registered general nurses	2 nurse aides
	3 midwives	1 environmental health officer
		1 dispensary assistant
Mission clinic	4 registered general nurses	3 nurse aides
Total	17	7

In addition, there were only 13 nurses, one environmental health officer and two nurse aides at the municipal clinic. The local private sector and community health volunteers had become incapacitated and literally non-existent. Compounding these health personnel shortages were an increased disease burden due to immigration, unplanned and haphazard settlement, poverty and lack of basic amenities such as water and sanitation systems. In a bid to cope with these challenges, the Ministry of Health intervened and facilitated the construction of another municipal clinic in 2007. In addition, the Ministry of Health formulated the 2009 Human Resource for Health Policy that it implemented through the Human Resource for Health Strategic Plan between 2009 and 2014 towards reviving the local human resource for health system in this community [11, 12]. As a result, there was an increase in the total number of health personnel of all cadres to 101, excluding seven general medical practitioners and three sisters in charge. These figures are outlined in Table 2.

**Table 2:** Staff establishment at health facilities in Epworth between 2009 and 2014

Facility type	Managers	Nursing staff	Other cadres	Total
Mission clinic	1 sister in charge	2 primary counsellors, 6 registered general nurses and 2 primary care nurses	1 environmental health officer/technician and 4 nurse aides	15
Municipal clinic 1	1 sister in charge	11 registered general nurses, 6 midwives, 1 state-certified nurse, 3 primary care nurses, 2 primary counsellors	1 pharmacy technician, 3 laboratory scientists, 3 ambulance drivers, 1 environmental health officer and 11 nurse aides	42
Municipal clinic 2	1 sister in charge	13 registered general nurses	1 dispensary assistant, 1 environmental technician, 5 nurse aides and 1 pharmacy technician	21
Private clinic	1 general practitioner	1 registered general nurse, 1 primary care nurse	2 nurse aides	4
Private clinic	1 general practitioner	1 registered general nurse	3 nurse aides, 1 lab pathologist, 1 radiologist and 1 dental surgeon	7
Private clinic	1 general practitioner	1 registered general nurse, 1 midwife	2 nurse aides	4
Private clinic	1 general practitioner	2 registered general nurses	1 nurse aide	3
Private clinic	1 general medical practitioner	1 primary care nurse	0	1
Private clinic	1 general practitioner	2 registered general nurses	0	2
Private clinic	1 general practitioner		2 nurse aides	2
Total		56	45	101

In this, there was an increase in the total number of health personnel to 15 at the mission clinic, 63 healthcare workers at the two municipal clinics and 38 at the private clinics. Epworth had a physician-to-patient ratio of 0.08:1000. This ratio included the seven general medical practitioners who operated private clinics in the community and six programme-specific voluntary medical doctors from the international non-governmental organisation (NGO). It appears that this was a notable increase because this physician-to-patient ratio compared favourably against the ratio of 0.06:1000 in the rest of Zimbabwe in 2009. However, the nurse-to-patient ratio of 0.35:1000 of 2014 compared less favourably to that of 1.3:1000 for the rest of Zimbabwe in 2011. Despite improvements in numbers, this implied a shortage of nurses which still persisted in this community by the end of 2014. These shortages manifested by way of a heavy workload of healthcare personnel, particularly at the two municipal clinics and one mission clinic. Because these numbers fell way short of requirements, strategies were implemented to retain health personnel recruited into this local system towards filling the shortage gap. However, little is known about health personnel retention strategies in peri-urban communities and their implications towards the human resource for health reform agenda of post-2008. Thus, we sought to explore the retention strategies of 2009 to 2014 in Epworth, a peri-urban community in south-east Harare. Our study sought to determine the strategies used to retain healthcare workers in Epworth between 2009 and 2014. We also sought to determine the implications of the retention strategies on other peri-urban communities throughout the world, towards the post-2008 human resource for health reform agenda.

## Methods

### Research design and study area

**Fig. 1:**
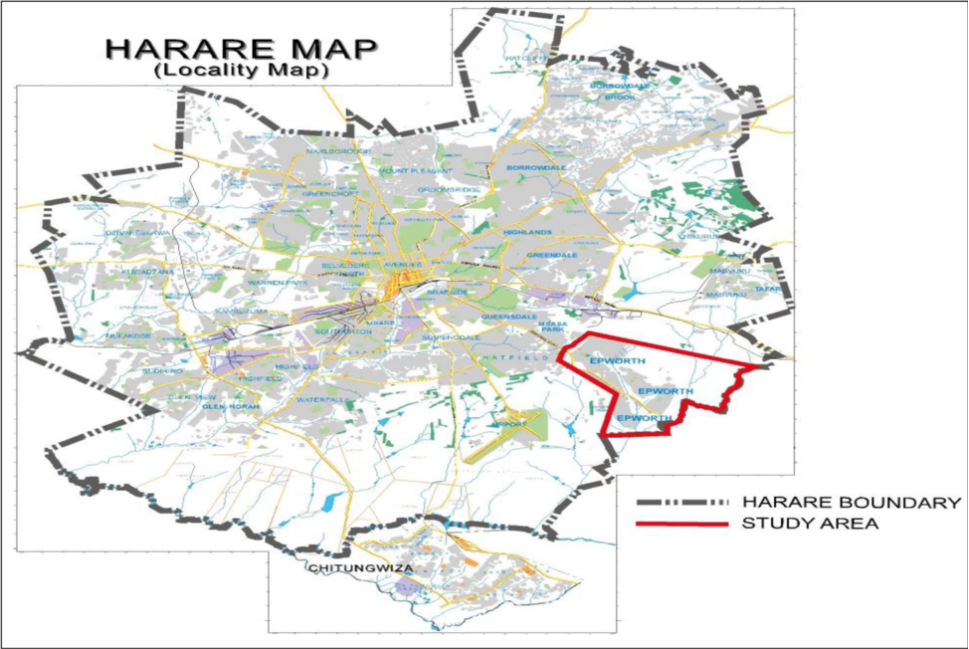
Location of Epworth on the Harare map

The research design was a cross-sectional survey, in which qualitative methods were used in sampling, data collection, reporting and analysis [13–15]. This study was carried out in Epworth, a peri-urban community on the south-east edge of Harare [16]. The location of Epworth is highlighted in the map of Harare in Fig. 1.

**Fig. 2:**
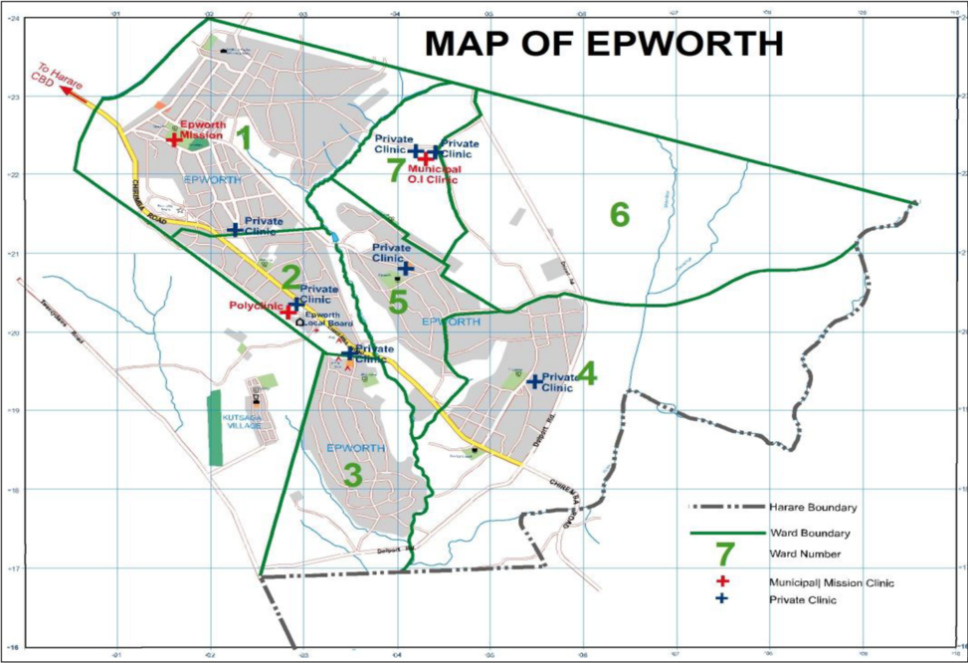
Map of Epworth. The map shows the locations of three public clinics which are represented by *red crosses* and seven private clinics represented by *blue crosses*. It also outlines the location of the seven wards that make up Epworth

Epworth consisted of seven wards as outlined in Fig. 2. In these seven wards, there are seven private clinics at places indicated by blue crosses. In addition, there are two municipal clinics and one mission clinic, indicated by red crosses on the map. It is through these clinics that human resource for health policy interventions were implemented between 2009 and 2014.

### Sampling methods

Purposive sampling was used to select health personnel at each of the 10 local clinics until saturation was reached [15]. The health personnel included registered general nurses, state-certified nurses, midwives, environmental health technicians and nurse aids. Purposive sampling was also used to select community health volunteers who participated in focus group discussions. They were placed into two main groups, namely community health workers/village health workers and peer educators, within which they participated.

### Data collection

At each clinic, an in-depth interview was carried out with a purposively selected sister in charge/human resource for health manager. Data was collected using interview guides, a pen and an audio digital recorder. Survey interviews were carried out with purposively sampled health personnel using an interview guide and pen until saturation was reached. Two focus group discussions were carried out, one with community health workers/village health workers and the other with peer educators. All healthcare workers who were approached provided written informed consent and participated in the study [14, 15].

### Institutional and ethical approval

This paper is an extraction from the main author’s PhD in public health research in which the aim was to determine how national human resource for health policy interventions impact local human resource for health systems using decision space mapping analysis. Research focused on Epworth, a peri-urban community in Harare, Zimbabwe. Institutional approval was granted by the Ministry of Health and Child Care of Zimbabwe, Health Services Board, Mashonaland East Provincial Medical Directorate, Seke District Medical Office, Epworth Local Board and Zimbabwe Republic Police. It was also approved by the Academic Advisory Committee of the University of Pretoria. Ethical approval was received from the Research Ethics Committee of the Faculty of Health Sciences, University of Pretoria (Reference number 413/2014). After this, ethical approval was granted by the Medical Research Council of Zimbabwe (Approval Number MRCZ/A/1941).

### Data analysis

Qualitative data collected was transcribed and examined to identify patterns across data sets. The identified patterns were then coded manually and subjected to thematic analysis from which conclusions were then reached [13–15].

## Results

### Healthcare worker retention strategies in Epworth

#### Engaging international partners through a programme-specific partnership

In the context of the Zimbabwe United Nations Development Assistance Framework (ZUNDAF), the Ministry of Health and Child Care entered into a programme-specific strategic partnership on HIV/AIDS, TB and malaria with an international non-governmental organisation (NGO). In its early days, this partnership facilitated the opening of the second municipal clinic in September 2011 and recruitment and deployment of more health personnel at both municipal clinics. In addition, it arranged to pay allowances for all health personnel for 2 years, whilst enabling gradual transfer of the responsibility to the Ministry of Health, which was also getting back to its feet during the same period. The 21 health personnel deployed at the second municipal clinic and 20 deployed at the other municipal clinic helped spread responsibilities which had a reduction effect on the workload. This also had positive implications towards retention as working conditions somewhat improved.

### Mobilisation of the community to assist health personnel at the clinics

Community health volunteers were recruited, trained and deployed to provide assistance to medical and non-medical staff at the two municipal clinics and one mission clinic. These consisted of peer educators and ward-based community health volunteers and village health workers. Peer educators were recruited from amongst HIV-positive members of the community. Training was provided in the context of the strategic partnership and focused on the provision of non-medical assistance to support service delivery at these clinics. Amongst their functions included assisting in patient’s file management. In addition, the organisation also recruited, trained and deployed ward-based community health workers. These were an addition to ward-based village health workers who had been recruited, trained and deployed into wards by the Ministry of Health and Child Care. The role of these ward-based community health volunteers was to assist in outreach activities that included the identification of unregistered pregnancies, basic home-based care, patient follow-ups and reporting. This impacted positively towards reducing the workload on health personnel at these facilities, hence also contributing towards retention. Apart from this, the Ministry of Health provided support for health personnel to enrol for post-basic training through the provincial and district medical offices.

### Supporting health personnel in post-basic training and development

The opportunity to enrol for post-basic training provided a sense of empowerment which contributed towards satisfaction and retention. Support was provided through a study leave on full salary and a tuition fee waiver to enrol for a Diploma or Higher National Diploma course or post-basic specialist course such as midwifery. Some respondents had a positive view towards this idea. One respondent expressed appreciation for the support received from the district medical office (DMO) when they stated the following:

The District Medical Officer is very supportive towards our enrolment for post-basic and postgraduate training. Whenever a training opportunity is announced by the Provincial Medical Directorate, the District Medical Officer is quick to let us know. They also facilitate the application and enrolment process to whoever would have been nominated to undertake these studies. Nomination for further training, mainly in Midwifery, is based on seniority which means that it is determined by how long you would have worked at this facility. One will continue to receive their salary whilst on study leave which is good. One only needs to be patient to wait for their turn to come before going for training. As we speak, two of my colleagues are away on study leave. They have enrolled for a course in Midwifery at Parirenyatwa School of Nursing. All this gives hope that eventually we will all get a chance to enrol for post-basic training.

However, other respondents had a different opinion on this. They were concerned and discouraged that the opportunity to enrol for post-basic training was limited and took much longer than they desired and expected. This was compounded by what they felt were very limited opportunities to enrol for post-basic training in subject areas of their choice. It appeared that this had negative implications towards their retention. This was expressed by one respondent who stated the following:

I am a Registered General Nurse but intend to enrol for a BSc Honours Degree in Environmental Health because the hierarchy in the nursing field is not flexible as the opportunity for further training is based on seniority. The District Medical Office selects people for post-basic training on the basis of seniority. As a result, it takes longer to receive that opportunity if you are junior and new. You are forced to wait for 7; 8 or 9 years before you receive an opportunity to enrol for post-basic training. This is compounded by the fact that the limited opportunities will force you to enrol for a specialist course in an area that is not of your choice. You are forced to do what they require, such as midwifery. It seems that these older ladies feel threatened by your pursuit of education ad want you to specialise only on the services available in the district and nothing else. You are forced to take up what they offer and they ignore your needs as an individual. I want to do a Degree in Environmental Health. If they do not want me to offer that opportunity then I will have to do it in a different capacity elsewhere and not as a nurse here. No one listens to you and there is no forum to discuss issues and lay out our grievances regarding career advancement.

However, the option to pursue post-basic training in an area of one’s choice appeared difficult for health personnel. Financial resource constraints, limited time and lack of support were viewed as major hindrances. There were limited tuition fee payment options for health personnel who wished to pursue academic degree programmes as the universities required payment in advance. This left a prospective candidate with the option of applying for a government cadetship. However, despite being limited, this option required that one be bonded for a certain period afterwards. Health personnel viewed this condition as unfavourable. Another option was for one to seek alternative sources of funding such as study loans. However, low salaries made this option impossible. This was compounded by the health personnel shortages and a heavy workload which made it next to impossible. One respondent summed it all up as follows:

I was offered a place to study for a Degree in Development Studies by one university but I do not have the financial resources to meet the cost. My wish is for the government to provide financial assistance to enable me to enrol for these studies. I must not fear furthering my studies in an area of my choice because in the end I will be providing services to the country. Another hindrance is the heavy workload at this clinic which makes it impossible for me to study and work at the same time. As you observed this morning, I had very long queues of patients who required attention. That is how I work each day. The government should assist me with a paid study leave to enable me to further my studies. After that, they should deduct whatever tuition they would have paid from my salary to reduce the bonding period.

Further compounding this was the effect that the awarding of a study leave would have on the other health personnel at the clinics. This concern was raised by health personnel as follows:

We are already seriously short staffed such that when one goes on study leave it becomes worse. It means that one would have to take up the workload left by whoever would have gone on training such that you end up covering a whole department meant for three healthcare workers alone.

To address this situation, the Ministry of Health provided local health personnel with on-the-job development opportunities. These were provided to health personnel at the two municipal clinics and one mission clinic through regular training workshops. These training workshops enabled healthcare workers to keep up with developments on various aspects of healthcare which included rapid HIV testing, malaria and TB management, sexual and gender-based violence, screening for TB, antiretroviral treatment and STIs, cancer screening and male circumcision. In addition, they also included prevention of mother-to-child transmission (PMTCT), paediatric and adult opportunistic infections (OIs), mentorship, IMAI-IMPAQ, nutrition management, energy planning, adherence counselling, multidrug resistance for TB, health promotion and advocation, kaposi sarcoma and monitoring and evaluation of health services. This also enabled them to stay ahead of patients, who enjoyed increased access to health information through the internet. On this, one respondent stated the following:

The training workshops which we attended were good for our work because they gave us knowledge which instilled confidence in what we do. However, refresher training courses are still necessary to enable us to keep up with developments on health issues and stay ahead because sometimes you find that, due to technology and the spread of internet onto cellphones, patients have more knowledge that you the service provider on a particular aspect of health and it can be very embarrassing to be in that situation. In addition, refresher courses are necessary because health and medical practice are dynamic and ever changing.

For health personnel at the private clinics, opportunities to enrol for post-basic and post-graduate qualifications depended on their employers. It appeared that the decision to enrol was a sacrificial choice between losing their job and possibly becoming unemployed thereafter or keeping it without any further training. Respondents in this category expressed dissatisfaction and the need for post-graduate training. For instance, one nurse at a 24-h private clinic stated that it was difficult for them to assist some patients that often came to their facility in labour during the middle of the night. Some nurse aids, however, stated that they lacked training on health altogether and were unable to handle patients in the absence of a qualified nurse or medical doctor. From this, it appeared certain that health personnel in this category were more difficult to retain because of very limited and literally non-existent post-basic training opportunities, which also negatively affected their ability to provide certain services. As a result, the lure of greener pastures elsewhere appeared overwhelming and inevitable.

### Salary review and payment

The Ministry of Health and Child Care intervened through a review and payment of salaries for health personnel at the two municipal clinics and one mission clinic. To start with, remuneration packages were reviewed in 2009 to denominate them in United States Dollars because the local currency had lost its value due to high inflation. This also followed the introduction of the multicurrency regime which legalised the use of foreign currency denominations in Zimbabwe. Prominent amongst these were the United States Dollar and South African Rand. This had the effect of bringing about financial certainty to healthcare workers because the Zimbabwean Dollar that had been used before had become uncertain and unstable due to high inflation as noted by one respondent:

The local currency had become uncertain due to high inflation. It was such that you would get paid one day but not afford transport to come to work the next day. It was a very difficult period. However, after 2009, things got better for us as we started receiving our salaries denominated in the US Dollar. Since then, things have stabilised and you can now budget even though the money is not enough to meet requirements.

In this context, the salary levels were determined by the Ministry of Health and Child Care through the national budgetary allocation from the Ministry of Finance and Economic Development. Decision-making on this was not decentralised to the local board between 2009 and 2014. In addition to this, arrangements were made to prioritise the payment of salaries without any delays each month. However, despite the fact that their salaries were comparatively higher than those for healthcare workers at the seven private clinics located in the community, and there being no delays in their payment each month, most healthcare workers indicated a lack of satisfaction with their basic income. Amongst this category was one respondent who stated the following:

My monthly salary is not enough to meet basic expenses such as transport, food, clothing and school fees for my children. The mission used to pay school fees for our children but it has since stopped and things are getting difficult for us. It would be better if the monthly salary was increased to at least US$ 1 000. Also, our colleagues working in the private sector in other places earn US$ 1 200 per month, whilst those at the international NGO working in this community earn US$ 1 500 per month. In that context, our lower salaries are demotivating.

Some were not happy that the transition from having their salaries paid by the international NGO to the government had resulted in a reduction in their salary.

My salary was reduced when the international NGO stopped paying and handed us over to the Ministry of Health. Since then, my salary became lower.

Another respondent was concerned that the salary grading system did not reflect on differences in qualifications, in addition to factors such as work environment and work load.

The grading system must be reviewed because currently a Nurse earns slightly a higher salary than a security guard. This is not fair at all considering differences in the level of education. Compounding this is that a Nurse with a university degree and the other with a diploma fall in the same salary grade. This makes it seem as though there is no benefit in furthering studies after all. There should be a beneficial solution regarding the determination of pay on the basis of education. Salary grades must be based on the level of education, and should also be based on the state of the working environment and the workload associated with the number of patients one serves during a specific period.

Some respondents in this category indicated that their salary did not match their expectations because of the risk of contracting diseases whilst providing treatment to patients. Some indicated that this left them in a state of stress and depression. On this, one respondent raised their concern in the following manner:

I work in the TB Department where I have a higher risk of contracting the disease but there is no compensation in the event that I contract MDRTB. There is also no guarantee that I will keep my job afterwards because they only give 90 days of sick leave yet it requires between 6 to 7 months, or even 2 years of treatment. During this period I will be infectious to patients but yet expected to be on duty. There is no policy to cover health cadres from TB in the event that you contract it on duty. Compounding this is that it is highly likely that I will contract it in the TB department that I work in. It makes me very scared because in the event that I contract the disease, there is no security for me from the Ministry of Health despite the fact that I am sacrificing myself to serve the nation.

A few respondents revealed having secondary employment at private clinics in other communities around Harare where they worked on a part-time basis after hours, during weekends and their off days to help supplement their monthly income. Some also revealed taking anti-depressants to cope with the stress associated with a low salary, heavy workload and a high-risk environment. Others revealed plans to apply for nursing jobs in other countries in the region and overseas whilst a few indicated plans to enrol for university degree programmes to change their profession all together.

### Payment of top-up allowances to supplement salaries

To help address the situation, it was established that the local board and the Methodist Mission complement central government effort towards retaining healthcare workers. The local board intervened through payment of a top-up allowance to health personnel at the two municipal clinics, whilst the mission did the same for some of its workers. It was established that the amount was about a quarter of their monthly salaries and that it was paid during the middle of each month and was separated from their monthly salaries. In addition, it was also established that there was a donor allowance paid to all health personnel of these facilities. Whilst the payment of this donor allowance was inconsistent, the amount paid was also very low and varied each month. Almost all health personnel interviewed expressed satisfaction with this arrangement. They stated that it helped supplement their salaries. For others, the fact that the allowance was paid during the middle of each month had helped them towards meeting transport costs because their salary would have run out at that time. The researcher also observed a sense of excitement amongst healthcare workers each time the top-up allowance was mentioned. On this, one respondent stated the following:

The top-up allowance that we received from the Local Board helped supplement our salary. This is because I received this payment during the middle of the month, time by which my salary would have run out. As a result, I am able to meet basic expenses such as transport until my next salary is paid. However, it should be increased to about $300 per month so that I am able to sustain my family for the whole month. We also get a donor allowance but the payment of this money is not consistent and its amount is much lower than what we are getting from the Local Board. Sometimes it is just $20 whilst at other times it is $50. At least it helps whenever it comes because the donor pays it when we do not expect it.

It was, however, established that some of the health personnel at these facilities did not receive the top-up allowance, particularly those that were deployed between 2009 and 2014. This created a strong sense of exclusion and division amongst this category of healthcare workers as expressed by one who indicated the following:

Not all of us receive those top-up allowances. Those of us who started working here in 2013 are not receiving those top-up allowances. For me this it is demoralising and I cannot get it out of my mind when working because we do the same job and serve the same patients but yet treated differently. Also, you find that someone might even be away on study leave whilst also receiving the top-up allowance whilst you are on duty each day but getting nothing. That is not fair at all. It hurts and makes you feel unwanted, unappreciated and less special than those that are getting. The other thing is that I am a Registered General Nurse but then you find that a Nurse Aid is receiving the top-up allowance whilst I am not. It hurts each time they call some from amongst us working in the same consultation room to go and receive those top-up allowances. We have tried engaging them on the matter but they seem reluctant to respond. I am not happy about this at all.

### Non-monetary long-service benefits

The local board provided free residential stands to health personnel as a form of long-service benefit. It was established that those that had worked at the two municipal clinics for at least 5 years received this benefit.

I was allocated a free residential stand as a long service award from the Local Board. The Local Board rewarded me for completing at least 5 years of service at this clinic. I am happy because I did not have to pay a cent for it.

The prospect of a free residential stand provided other health personnel with a sense of hope that they too would one day receive them. This had positive implications on their commitment to stay in their job.

### Responsive support in the provision of medical supplies and sundries

Health personnel also expressed appreciation in the responsive support they received from the local board towards the provision of medical supplies and sundries. Responsive support made their work easier as stated below:

The Local Board supports our work here at this clinic. They are very responsive towards our needs. If we request sundries such as vim, soap and methylated spirit, they deliver on time which means that there will be no strain for us in delivering services. Whenever we prepare our paperwork on time, they also respond timely.

The provision of support helped reduce the strain of work on health personnel at the municipal clinics. These clinics were often busier and congested by locals. This had positive implications on retention.

### Protocol to protect health personnel from communicable diseases

There were clinical procedures and standards to protect health personnel from accidentally contracting diseases. Amongst these were Prophylaxis Guidelines, a protocol outlining procedures to be followed when handling HIV/AIDS patients and procedures to be followed in the case of an accidental exposure. Health personnel stated that these protocols were necessary and helpful considering that their work environment was associated with high risk and pressure. A respondent at the one of the municipal clinics indicated that the protocol helped them to remain HIV negative.

We are different from other facilities in the sense that 90% of the patients I see each day are HIV positive such that during testing, injection and collection of blood samples I am always at a high risk. For example, one day I had drawn blood samples from a minor to check their CD4 count. However, the child accidentally kicked me which resulted in the syringe that I had used accidentally piercing into my skin and getting the sample into my exposed arm. I had to go on prophylaxis since the child was HIV positive. These risks are part of my daily life. If there was a risk allowance maybe it would help ease my mind. I feel different from other healthcare workers who go through what I experience each day only once in 10 years at their facilities. I am at greater risk.

However, whilst the guidelines were beneficial, it appeared that enforcement was undermined by lack of training and enforcement amongst non-medical health personnel. On this, one non-medical health worker narrated how they contracted HIV whilst performing their duties in an emergency situation at one of the clinics:

I work in the Maternity Ward as a Nurse-aid. Before this, I worked at this clinic as a volunteer. I divorced from my husband in 2000 and have never been in another sexual relationship since. Since my divorce, I have always tested for HIV to ensure that my husband had not infected me before we separated. Well, I was happy to find out that I tested negative each time. Sometime in September-October 2012, I worked on a night shift in the maternity ward. There was no electricity and our generator was not working. That same night, a pregnant woman who was not booked approached our ward and was already in labour. By the time we attempted to attend to her in the corridor by the entrance, the baby started coming out. It became an emergency situation in which there was no time to take her into the ward. This forced us to assist the patient in having the delivery take place in the corridor before taking her into the ward. However, when this woman was separated from her newly born child, everything that had been used during the delivery was put into the same receiver. Immediately afterwards, as I separated the utensils from the other material for discarding, I failed to notice that the surgical blade that had been used to cut the umbilical cord had been left in the receiver where it was not supposed to be in the first place. This blade was supposed to have been disposed into the sharps litter box immediately after use. As I started washing the receiver in the dark, I accidentally touched the surgical blade because I had not seen it. As a result, I suffered a deep cut from which I started bleeding. I washed my finger on running water before applying some medicine and dressing it with a bandage. Two days later, I completed my night shift before going off duty for the nine days that followed. It was only after I had returned to work on a day shift when I established from my colleagues that I was supposed to have gone through the Prophylaxis Protocol within 72 hours to prevent HIV infection. Unfortunately it was too late at that point, and I did not have any prior knowledge about this protocol. A few months later, I tested positive for HIV. This is the only source of my infection that I know. Nevertheless, I love my job and continue executing my duties in the Maternity Ward. I recommend that these guidelines be used in all situations no matter how small the incident seems. Further, I also recommend that guidance be provided to all health personnel about them. At least I am glad to have shared this experience with you. This has set me free because I had not been able to tell anyone about it before.

Despite the passion to continue in light of this negative development as narrated above, one cannot doubt the negative implications that this has on the retention of other volunteers and non-medical personnel.

## Discussion

The review of salaries was identified as the first theme. The Ministry of Health intervened towards retention through salary payment for health personnel at the two municipal clinics and mission clinic. Remuneration packages for all healthcare workers were reviewed in 2007 by denominating them in United States Dollars because the local currency had lost its value due to high inflation. This was followed by the introduction of the multicurrency regime which legalised the use of foreign currency denominations in Zimbabwe, prominent of which were the United States Dollars and South African Rand. Whilst this had the effect of bringing about financial stability to healthcare workers, it created another challenge which threatened their retention. Health personnel were not satisfied with their salary levels because they did not meet basic needs such as transport, accommodation, food, clothing and school fees for their children. This was compounded by the expectation for higher remuneration based on a heavy workload, pressure and risk of disease infection. Further compounding this was that income levels did not reflect the differences in education levels because salary grades for a nurse qualified with a university degree and the other with a diploma were the same. This was made worse by the slight difference between the salary levels of a security guard and a nurse, despite the bigger difference in their levels of education. As a result, a few revealed having secondary employment at private clinics in other communities around Harare where they worked on a part-time basis after hours, during weekends and during their off days to help supplement their monthly incomes. Some also revealed taking anti-depressants to cope with stress whilst others revealed plans to apply for nursing jobs in other neighbouring and overseas countries and/or enrol for university degree programmes to change their profession all together. In a bid to overcome these challenges, the Ministry of Health engaged international strategic partners for supplementary effort towards overcoming these challenges. Accordingly, our second theme focuses on this intervention.

The Ministry of Health engaged an international NGO to help supplement government effort towards health personnel retention. This strategic partner was engaged in the context of ZUNDAF to help retain health personnel, towards reviving the local human resource for health system [11]. Strategic partnerships with the donor community have always been used to overcome health personnel challenges in developing countries. In 2005, the Malawi government, with support from donors, initiated the 6-year Emergency Human Resources Programme to alleviate the human resource crisis in health districts. The key components are a salary increase for health professionals; measures to enhance the capacity of training institutions; and, in the short term, additional recruitment of expatriate volunteer doctors and nursing tutors. Of the three components, the salary top-up scheme is designed to improve the working conditions for existing staff and aims to increase retention of health workers in public service [17–19]. Our results also suggest that a programme-specific strategic partnership between the Ministry of Health and an international NGO was used to retain healthcare workers between 2009 and 2014. To increase remuneration for health personnel as was done in Malawi, the NGO initially paid allowances to all health personnel at the two municipal and one mission clinic between 2007 and 2009. The mandate to pay salaries remained with the government. However, between 2007 and 2009, this mandate was undermined by high inflation experienced during that time which made government salaries irrelevant. This literally meant that health personnel were sustained by allowances paid by the donor organisation which were denominated in foreign currency until 2009 when the responsibility was handed over to the government. This contributed towards their retention, which also facilitated the revival of the local human resource for health system. Salaries were supplemented by the payment of top-up allowances. Our third theme focused on this strategy.

Top-up allowances were paid to help supplement the salaries paid to health personnel at the two municipal clinics and mission clinic in Epworth. They were paid by the local board, mission and a donor. However, donor intervention was less visible and inconsistent as health personnel at these three facilities indicated that the top-up allowance paid by the donor was inconsistent and comparatively much lower. Similar experiences were shared with Malawi where the salary top-up scheme contributed towards the improvement of working conditions for existing staff and retention of health workers in public service [19]. In Epworth, however, it was established that not all health personnel received the top-up allowance payment. A small minority of health personnel at the first municipal clinic and the majority at the mission clinic did not receive these top-up allowances. This created a sense of exclusion and division amongst health personnel. In addition, this also undermined morale amongst those who did not receive this top-up allowance. Compounding this was the expectation for a higher top-up allowance amongst those that received this top-up allowance. In addition to the unsatisfactory salaries, this had negative implications towards the retention of health personnel at these facilities. Apart from this, training and development also contributed towards the retention of health personnel.

We identified the support towards post-basic training and development as our fourth theme. Training and development interventions helped instil a sense of empowerment amongst health personnel in Epworth which contributed towards their retention. Training and development interventions were also used in Malawi where donor assistance through the 6-year Emergency Human Resources Programme helped to alleviate the human resource crisis in the health sector through building the capacity of training institutions [19]. In contrast, however, there were no training institutions in Epworth. Capacity building in this area was pursued through the provision of support to health personnel towards their enrolment for post-basic training. However, opportunities appeared few and far in-between which had negative implications towards the retention of health personnel. To help counter this, the Ministry of Health engaged its strategic partners towards the provision of on-the-job development through regular training workshops on various aspects of health. These helped health personnel to stay up to date with developments on various aspects of health. In addition, the prospect of attending these regular workshops had a positive effect towards the motivation of health personnel. It provided a sense of empowerment, apart from being just a source of temporary reprieve from their busy work environment. This had positive implications towards their retention.

However, we also established that there were mixed views regarding support to pursue post-basic training amongst health personnel. Whilst others had a positive view towards the support by the district medical office, others had different opinions. Some respondents expressed concern and discouragement regarding the few and far in-between opportunities to receive support to enrol for post-basic training. This was compounded by what they felt were very limited opportunities to enrol for post-basic training in subject areas of their choice. This was also compounded by financial resource constraints, limited time and lack of support which were viewed as major hindrances to pursuing training in areas of one’s choice. Apart from this, some were concerned about a heavier workload due to shortages emanating from colleagues who would have been awarded a study leave. It appeared that all these views had negative implications on the retention of health personnel.

Our fifth theme focused on the strategy to reduce the workload through the mobilisation of more personnel. The mobilisation of additional personnel was undertaken towards reducing the burden of a heavy workload on the few available healthcare workers. Initially, the international NGO recruited and deployed 20 health personnel to complement the local staff complement of 17 that existed at the first municipal clinic in 2007. In addition, it also facilitated the opening of the second municipal clinic at which it facilitated the deployment and remuneration of 21 health personnel during the same period. The opening of the second municipal clinic in 1997 helped reduce pressure on the first municipal and the mission clinic. This had positive implications towards healthcare worker retention. In addition, this also allowed for the Ministry of Health to gradually take over some of the health personnel from the international NGO and deploy others. As a result, there were 25 more health workers in addition to the 17 that had existed at this clinic in 2007.

Apart from this, we also established in our study that the international NGO also recruited and deployed six programme-specific voluntary medical doctors to help in HIV/AIDS, TB and malaria interventions through the municipal clinic between 2009 and 2014. This helped ease the burden on nursing staff at this facility which also contributed somewhat to their retention. This was similar to interventions in Malawi where additional expatriate volunteer doctors were recruited in the short term by the donor. This helped reduce the burden on nurses in delivering healthcare services [19]. In addition, we also established that community health volunteers were recruited and deployed to help reduce the burden on health personnel at the two municipal and mission clinics. Community health volunteers were divided into two categories, namely peer educators and ward-based community health workers/village health workers. Peer educators provided assistance through the non-medical aspects of service delivery at the three clinics. This helped reduce the burden on health personnel at these clinics. We established that this outcome was similar to that of Ghana during the implementation of the Community-based Health Planning and Services Initiative [20]. In that intervention, the community health volunteers who operated from community health compounds provided assistance to the community health officer in performing on-site and outreach work. This helped reduce the burden on community health officers in the provision of healthcare services to the community [20]. We identified the provision of non-monetary incentives as our sixth theme.

The local board provided free residential stands as a long-service benefit for health personnel. We established in our study that health personnel who had worked at the municipal clinics for at least 5 years were allocated a free residential stand by the local board. This incentive created a sense of hope and expectation amongst health personnel. It appears that this had a positive effect towards the retention of healthcare workers. For health personnel at the local private clinics, the provision of non-monetary incentives depended on what their employers could afford to give. Apart from this, there were protocols to protect health personnel in a high-risk environment. This strategy was the seventh theme which we identified from this study.

There were clinical protocols and standards to protect healthcare workers from communicable diseases. Prominent amongst these were Prophylaxis Guidelines aimed to protect health personnel from accidental infection with HIV whilst executing their duties. They outlined the protocol to be followed when handling HIV/AIDS patients and treatment procedures to be followed in the case of an accidental exposure. We established in our study that these provided a sense of protection to health personnel which also significantly contributed towards their retention. However, whilst the guidelines had benefited some, other respondents were not fortunate to benefit from them due to lack of knowledge and assistance. The case of one nurse aid who did not receive assistance and advice on time after accidental exposure raises concern. It has an undermining effect on the extent to which non-medical volunteers will be willing to commit to the service in a peri-urban community in the future. Very few would have the courage and passion to continue providing services, sometimes without any remuneration, after having been infected accidentally in the same workplace. Compounding this was the high risk of contracting multidrug-resistant tuberculosis (MDRTB) which left health personnel from the TB departments in a state of worry. This was made worse by the fact that MDRTB required a longer period of treatment beyond the 90 days of sick leave. As a result, this made health personnel insecure about the prospects of keeping their job in the event of accidental infection, in addition to the fear of contracting it in a high-risk environment.

## Conclusions

It was concluded that the retention strategies implemented in Epworth between 2009 and 2015 contributed towards the revival of the local human resource for health system. This had positive implications towards human resource for health reform of the 21st century. The strategies implemented included a programme-specific strategic partnership between the government and donor community. This partnership contributed towards more healthcare workers, more health facilities, worker development and remuneration. In turn, this contributed towards reducing the workload on individual health personnel which had positive implications towards their retention. In addition, the Ministry of Health intervened through the review and payment of salaries and provision of support for post-basic training and development. To complement this, it was concluded that the local board and mission contributed through the payment of top-up allowances and provision non-monetary incentives. This also had positive implications towards the retention of healthcare workers during this period. However, it was also concluded that these interventions did not meet the expectation of all health personnel. This threatens to undermine the retention of health personnel, and has negative implications towards the human resource for health reform, and health system appraisal of the 21st century.

## Abbreviations

DMO: district medical office
MDRTB: multidrug-resistant tuberculosis
MRCZ: Medical Research Council of Zimbabwe
NGO: non-governmental organisation
OI: opportunistic infection
PMD: Provincial Medical Directorate
PMTCT: prevention of mother-to-child transmission
US: United States
ZUNDAF: Zimbabwe United Nations Assistance Development Framework
